# BODIPY Conjugate of Epibrassinolide as a Novel Biologically Active Probe for In Vivo Imaging

**DOI:** 10.3390/ijms22073599

**Published:** 2021-03-30

**Authors:** Anastasiia Starodubtseva, Tetiana Kalachova, Oksana Iakovenko, Vera Stoudková, Vladimir Zhabinskii, Vladimir Khripach, Eric Ruelland, Jan Martinec, Lenka Burketová, Volodymyr Kravets

**Affiliations:** 1 Institute of Experimental Botany of the Czech Academy of Sciences, Rozvojová 263, 165 02 Prague, Czech Republic; starodubtseva@ueb.cas.cz (A.S.); iakovenko@ueb.cas.cz (O.I.); stoudkovavera@seznam.cz (V.S.); martinec@ueb.cas.cz (J.M.); burketova@ueb.cas.cz (L.B.); 2 Department of Biochemistry and Microbiology, University of Chemistry and Technology, Prague, Technická 5, 166 28 Prague, Czech Republic; 3 Institute of Ecology and Environmental Sciences of Paris, Paris-Est University, UPEC, 94010 Créteil, France; 4 V.P. Kukhar Institute of Bioorganic Chemistry and Petrochemistry, National Academy of Sciences of Ukraine, 02094 Kyiv, Ukraine; kravets@nas.gov.ua; 5 Institute of Bioorganic Chemistry, National Academy of Sciences of Belarus, Kuprevich Str., 5/2, 220141 Minsk, Belarus; vz@iboch.by (V.Z.); khripach@iboch.by (V.K.); 6 UMR 7025 CNRS, GEC Génie Enzymatique et Cellulaire, Centre de Recherches, Rue Personne de Roberval, CS 60319, Alliance Sorbonne Universités, Université de Technologie de Compiègne, 60203 Compiègne CEDEX, France; eric.ruelland@utc.fr

**Keywords:** brassinosteroids, fluorescent conjugates, plant bioassay, live imaging

## Abstract

Brassinosteroids (BRs) are plant hormones of steroid nature, regulating various developmental and adaptive processes. The perception, transport, and signaling of BRs are actively studied nowadays via a wide range of biochemical and genetic tools. However, most of the knowledge about BRs intracellular localization and turnover relies on the visualization of the receptors or cellular compartments using dyes or fluorescent protein fusions. We have previously synthesized a conjugate of epibrassinolide with green fluorescent dye BODIPY (eBL-BODIPY). Here we present a detailed assessment of the compound bioactivity and its suitability as probe for in vivo visualization of BRs. We show that eBL-BODIPY rapidly penetrates epidermal cells of *Arabidopsis thaliana* roots and after long exposure causes physiological and transcriptomic responses similar to the natural hormone.

## 1. Introduction

Brassinosteroids (BRs) are plant hormones of steroid nature. The most abundant are brassinolide and castasterone, produced in various plant organs and acting mostly in the neighboring cells and tissues [1,2]. Synthesis, transport, and signaling pathways of BRs are being actively studied, but gaps remain in understanding the mechanisms of BRs action on a cellular level. The physiological effects of brassinosteroids rely on specific recognition of the compound by a complex including leucine-rich-repeat receptor-like protein kinases (LRR-RLKs) BRI1 [3] and BRI1-associated receptor kinase 1 (BAK1) [4], which in turn initiates an intracellular phosphorylation relay cascade [5,6,7]. BRI1 subsequently phosphorylates its inhibitor BKI1 (BRI1 KINASE INHIBITOR1) and induces its dissociation from the plasma membrane (PM) [4,8,9], thus enabling heterodimerization, reciprocal phosphorylation, and full activation of BRI1 and BAK1 kinases [10,11]. BRI1 phosphorylates BSK1 (BR-SIGNALING KINASE1), CDG1 (CONSTITUTIVE DIFFERENTIAL GROWTH1), and some of their homologs, leading to the activation of BSU1 (BRI1 suppressor1) and BSU1-Like1-3 (BSL1-3) [12,13,14]. BSU1/BSLs then inactivate BIN2 (Brassinosteroid-Insensitive2) [15]. As a result, BIN2 substrates BZR1 (BRASSINAZOLE-RESISTANT1) and BES1 (BRI1-EMS SUPPRESSOR1) [16] get dephosphorylated and transported to the nucleus [17] where they target promoters containing BR-response element CGTGC/TG and/or E-box (CANNTG) motif to regulate the expression of thousands of BRs-responsive genes that are crucial for plant growth and development [18,19]. As a result, special groups of genes are being induced or repressed, modifying cell metabolism, and whole plant physiology. The effect of BRs on transcriptome is not restricted to a few specific genetic targets but affects genes associated with other hormone signaling pathways, especially salicylic acid (SA), abscisic acid (ABA), jasmonic acid (JA), and auxins [20].

The majority of the data about BR transduction have been obtained using molecular and genetics approaches targeting receptor proteins and/or signaling components. To untangle the complexity of BRs action *in planta*, the visualization approach can be of great use. For several plant hormones, conjugates with fluorescent dyes have been used with promising results. For example, biological activity has been reported for the conjugates of auxin [21,22], ABA [23,24,25], cytokinins [26] and jasmonic acid [27]. For brassinosteroids, a number of fluorescent conjugates have been synthesized [25,28,29,30,31,32,33,34,35,36]. These conjugates have been mostly used in vitro, in the context of BRs quantitation, by HPLC [34] or in immunosorbent assays [34,37,38]. 

However, the biological properties of such conjugates in vivo often differ from the original BRs. Recently Irani et al. developed a conjugate of castasterone with far-red-fluorescent Alexa Fluor 647 dye, AFCS, that has been successfully applied for studies of BRI1 receptor endocytosis [39]. AFCS has been proven bioactive based on root growth assay and effect on transcription of BRs biosynthetic genes. Still, the compound was active in micromolar concentrations, while natural BRs activity lies in the nanomolar range. Lower activity of AFCS can be due to the bulky fluorophore group that affects penetration through the cell wall and/or influences binding to the receptor. A common disadvantage of almost all the described brassinosteroid conjugates is that one or more functional groups determining their biological activity are used to bind with a fluorophore [28,29,34]. Attempts to overcome this drawback led to the development of a number of new conjugates [35,40]. Lately, Hurski and colleagues have reported the synthesis of a compound that seems to have high potential as a fully functional fluorescent conjugate of 24-epibrassinolide (eBL) with green-fluorescent dye BODIPY (eBL-BODIPY) [40]. In this compound, BODIPY dye is fused in a way that functional groups of eBL remain intact, while the fluorescent properties of the molecule are similar to that of BODIPY. In addition to preserving all functional groups, this conjugate uses BODIPY as a dye. It is believed that such dyes have a number of advantages over other fluorophores [41], including a high fluorescence quantum yield, a relatively long excited state lifetime, and a small dependence of the fluorescence properties on pH and polarity [42]. Here, we present the first assessment of the biological activity of eBL-BODIPY *in planta* and propose it as a probe suitable for localization studies by fluorescent microscopy. 

## 2. Results

### 2.1. eBL-BODIPY Affects Root Growth Similarly to eBL

**Figure 1:**
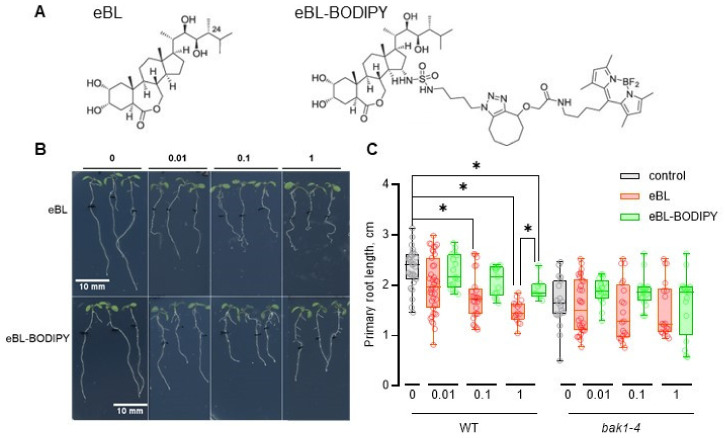
Epibrassinolide with green fluorescent dye BODIPY (eBL-BODIPY) affects root growth similarly to eBL. (**A**) Chemical structures of eBL and eBL-BODIPY; (**B**) representative images of 7-day-old wild type (WT) seedlings after 3 days of cultivation on solid ½ Murashige-Skoog medium containing various concentrations (μM) of compounds; scale bars 10 mm; black mark indicates root tip position at the moment of transfer; (**C**) primary root length, *n* ≥ 18; *p* < 0.05, * indicates significant differences between groups, one-way ANOVA.

As a model, we chose seedlings of *Arabidopsis thaliana* Col-0 (wild type; WT), one of the most used and well-characterized plant systems. First, we compared the effect of eBL and eBL-BODIPY on root growth elongation (Figure 1). To ensure that the observed effect is connected with the specific molecule recognition by BRI1-BAK1 receptor complex, as a negative control we used a T-DNA knock-out *bak1-4* mutant [43]. Four-day-old seedlings were transferred to Petri dishes containing various concentrations of brassinosteroids (0.01, 0.1, and 1 μM). Three days later primary root, meristem, and cortical cell length were then measured. As expected, eBL caused dose-dependent root shortening in WT plants, but not in the *bak1-4* mutant. Similarly, the presence of low concentrations of eBL-BODIPY caused root shortening in WT plants (Figure 1B). Notably, while roots of plants grown on eBL exhibited typical waving phenotype, this was not the case for eBL-BODIPY. We also observed shortening of the meristematic zone and cortical cells upon eBL in WT plants, but not *bak1-4* (Supplemental Figure S1). For this trait, eBL-BODIPY was found to be less efficient, however still significantly active at 0.1 μM and 1 μM concentrations.

### 2.2. eBL-BODIPY Associates with Plant Cell Wall and Penetrates the Cytoplasm

As the probe is to be used in vivo, we investigated eBL-BODIPY behavior in association with plant roots. First, we confirmed the dye stability in the cultivation media and upon illumination for up to 3 days. The spectrum and intensity of the fluorescence in roots did not differ whether the roots were transferred on freshly prepared agar plates or the plates after 3 days in the cultivation room (data not shown). This suggests that the dye is suitable for long-term application and physiological studies. 

**Figure 2:**
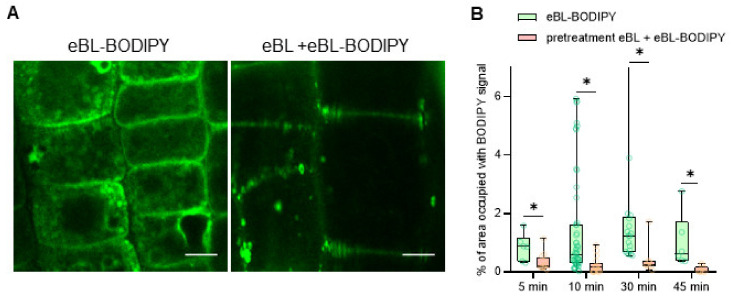
eBL-BODIPY internalizes into root epidermal cells. (**A**) Representative images of root epidermal cells after 30 min exposure to 10 μM of eBL-BODIPY (left panel: non-pretreated seedlings, right panel: seedlings pretreated for 60 min with 1 μM eBL), scale bars 5 μm; (**B**) quantification of the intracellular fluorescence in root epidermal cells exposed to 1 μM eBL-BODIPY for different time, *n* ≥ 20; * indicates significant differences between treatments within one sampling point, *p* < 0.05, *t*-test.

Next, we investigated kinetics and specificity of eBL-BODIPY intake on epidermal cells of apical root meristem. Five-day-old *A. thaliana* WT seedlings were incubated in a drop of 1 μM or 10 μM eBL-BODIPY, washed four times, counterstained with propidium iodide, and examined for intracellular BODIPY-associated fluorescence at distinct time points. Already after 5 min of eBL-BODIPY treatment, we observed accumulation of the fluorescent compound on the cell walls. As the exposure time increased, the signal appeared inside the cytoplasm. At 30 min, we observed a strong cytoplasmic fluorescence (Figure 2A, left panel). To test whether such internalization was caused by a specific interaction with brassinosteroid receptors, we performed a competition assay: seedlings were pre-incubated with eBL for 60 min prior to treatment with fluorescent conjugate. The pre-treatment inhibited signal internalization (Figure 2A,B), which can be explained by occupation of the receptor complex by eBL that prevented binding of eBL-BODIPY.

**Figure 3:**
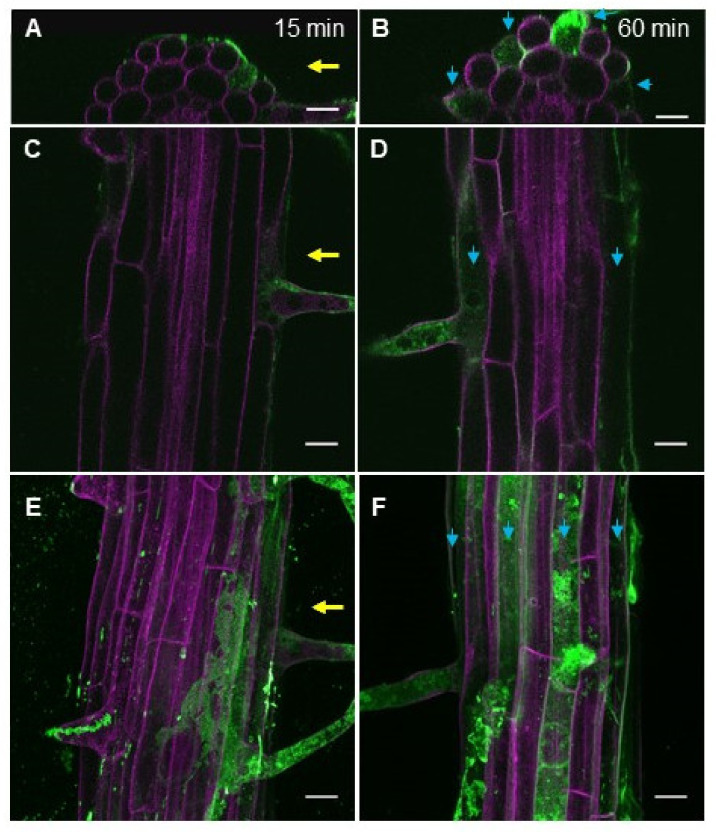
eBL-BODIPY rapidly aggregates with the cell wall and penetrates the epidermal cells. Five-day-old seedlings were placed on solid MS medium containing 0.1 μM eBL-BODIPY and observed after 15 min (**A**,**C**,**E**) and 60 min (**B**,**D**,**F**): orthogonal cuts of XZ (**A**,**B**) and XY (**C**,**D**) planes, maximum intensity Z-projections (**E**,**F**); membrane fluorescence (magenta) and BODIPY fluorescence (green). Yellow arrows indicate the root side directly exposed to eBL-BODIPY. Blue arrows point to trichoblast cell lines. Scale bars 20 μm.

**Figure 4:**
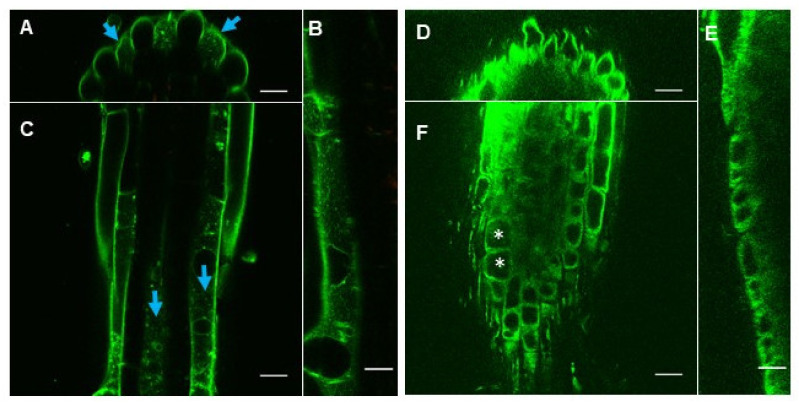
Representative images of differentiation zone (**A**,**B**,**C**) and meristem (**D**,**E**,**F**) of 5-day- old *Arabidopsis thaliana* Col-0 plants after 24 h of incubation on media containing 1 μM eBL-BODIPY. Orthogonal cuts of XZ (**A**,**D**), YZ (**B**,**E**) and XY (**C**,**F**) planes; Z-stack of 149 slices, scale bar 20 μm. Blue arrows indicate trichoblast cell lines; white asterisks indicate vacuolar space.

In the cell biology studies, fluorescent dyes for the imaging purposes are often applied on roots directly as drops, but in long-term physiological studies (i.e., root growth assays) the studied compounds are to reach cells from the media. We next examined eBL-BODIPY penetration into the roots placed in contact with dye-containing agar. In this setup, BODIPY fluorescence was detected in association with cell walls in the differentiation zone already after 15 min (Figure 3A,C,E). At this time point, the dye was aggregating around the root hair. Sixty min after the transfer, the fluorescence was also observed inside the cytoplasm of the epidermal cells (Figure 3B,D,F). Notably, only trichoblast cells accumulated the dye, suggesting that eBL-BODIPY penetration is rather happening via active trafficking than simple diffusion. Increasing the exposure time to 24 h and concentration to 1 μM resulted in higher fluorescence. However, the signal did not penetrate beyond the epidermal cell layer neither in the differentiation zone, nor in the meristem (Supplemental Figure S2, Figure 4).

In the epidermal cells of the meristem and differentiated trichoblasts, we observed vesicle-like aggregates in the cytoplasm (Figure 4F, Supplemental movie 1). No fluorescence was detected in vacuolar space or in the nuclei. All meristematic cells similarly incorporated the dye. However, no signal was detected inside the differentiated atrichoblasts even upon 24 h of exposure (Figure 4A–C). We have examined a range of eBL-BODIPY concentrations and recommend 0.01–0.1 μM and 30–60 min exposure time as optimal for imaging purposes.

After long-term dye application (3 days), the eBL-BODIPY localization in the meristematic zone remained smooth and cytoplasmic. In the differentiation zone, trichoblast cells showed strong cytoplasmic fluorescence and some BODIPY-positive vesicular bodies were observed in atrichoblasts. The high concentration of dye (1 μM) caused moderate toxic effects, as shown with dead cell nuclei counterstaining with propidium iodide (Supplemental Figures S2 and S3). We observed BODIPY-associated fluorescence all along the root and found it to be more intense in the differentiation zone than in the meristem or elongation zone. No fluorescence was detected in upper tissues that were not directly exposed to eBL-BODIPY.

### 2.3. eBL-BODIPY Partly Mimics Transcriptomic Response to eBL

**Figure 5:**
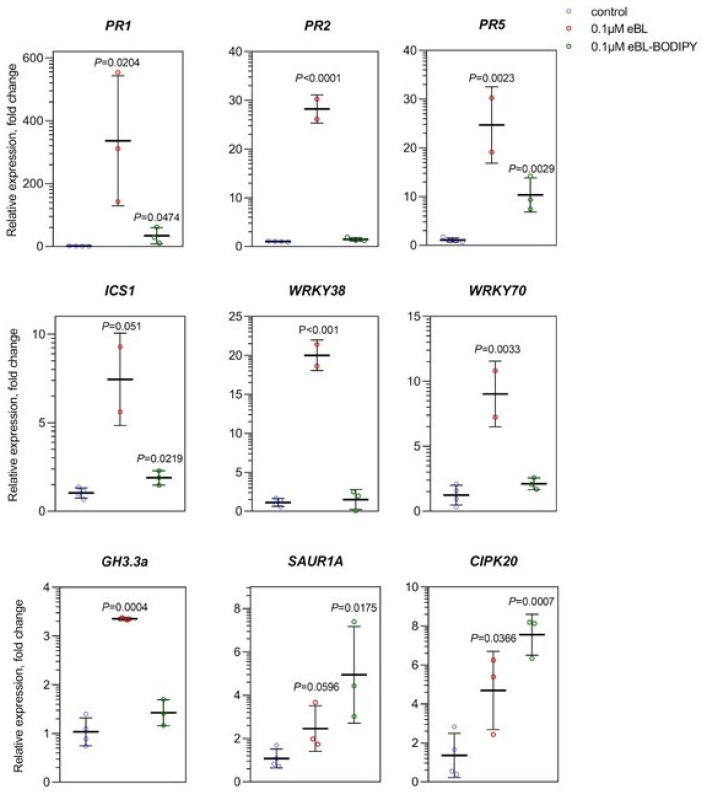
Effect of eBL and eBL-BODIPY on the transcription of selected genes, associated with salicylic acid (SA), auxin, and abscisic acid (ABA) responses. Five-day-old seedlings were exposed to 0.1 μM of eBL or eBL-BODIPY for 24 h prior to RNA extraction. The *p*-value indicated for variants significantly different from control, *t*-test, *n* = 3–4, *p <* 0.06.

To dissect the physiological response to eBL-BODIPY, we compared the effect of eBL and eBL-BODIPY on the transcriptome of Arabidopsis seedlings. Based on imaging and root growth assay results, we selected 0.1 μM and 1 μM concentrations and 24 h incubation time for analysis. Full seedlings were harvested for RNA extraction. Based on the published data of transcriptome changes upon BRs application [44], we selected genes responsive to BRs and involved BR biosynthesis (*DWF4*) and in the signaling cross-talk with other phytohormones: SA (*PR1, PR2, PR5, ICS1, WRKY38, WRKY70*), auxins (*GH3.3, SAUR1A, SHY2, AUX1*), ABA (*CIPK20, LEA4-1, ABI1*), and JA (*PDF1.2, LOX2*). Transcription of *DWF4* showed ca 30% decreased by 1 μM of eBL-BODIPY, comparable to a 40% decrease upon the action of eBL. However, 0.1 μM of eBL-BODIPY did not show significant impact on *DWF4* (Supplemental Figure S4). As for the genes, involved in the hormonal crosstalk, only the group of SA-related genes responded consistently to both compounds. However, the effect of eBL-BODIPY was less pronounced. For example, 0.1 μM of eBL caused ca 300-fold increase of *PR1* transcription, while 0.1 μM eBL-BODIPY only to a 30-fold increase) (Figure 5). However, 1 μM of eBL caused ca 5-fold increase of *ICS1, WRKY38* and *WRKY70* transcript accumulation, while application of eBL-BODIPY resulted in more than 10-fold induction (data not shown). That might signify moderate cytotoxicity of the compound, as *WRKY70* is associated with cell death in stress responses [45], often coupled with SA synthesis via ICS1 [46]. As for auxin-associated genes, *SAUR1A* and *GH3.3* were upregulated by both compounds in both studied concentrations, while accumulation of *SHY2* and *AUX1* was not changed in our system. Among studied ABA-associated genes, only *CIPK20* showed a significant increase in response to both eBL and its conjugate, 5-fold and 8-fold respectively. Transcription of JA-responsive genes was not significantly affected (data not shown).

## 3. Discussion

In this work, we assessed the biological activity and in vivo fluorescent properties of the previously synthesized eBL-BODIPY [40]. We showed that eBL-BODIPY rapidly penetrated cell walls (intracellular signal was detected 15–60 min after exposure to the molecule). This falls into a time-frame reported for another fluorescent conjugate, AFCS, which is recommended to be applied as a solution drop ca 40 min before root imaging [47]. eBL-BODIPY was effective at 0.1–10 µM concentrations, which is relatively high compared to the usual range of concentrations for physiological studies; however, up to 20 µM of AFCS (castasterone conjugate) have been suggested for short-term treatments for imaging purposes [47]. 

Remarkably, eBL-BODIPY actively penetrated only trichoblast cells, while no associated signal was detected in atrichoblasts (Figure 3 and Figure 4). The difference was drastic in differentiated cells, while non-differentiated meristematic cells responded similarly. The exact nature of this effect is yet to be discovered. However, it is in line with the studies pointing our special functions of BRs in different epidermal cell types. Hacham et al. have shown that both hair and non-hair cells have comparable BRI1 receptor densities on the plasma membrane and induce a similar increase in the transcript levels of *ACS5* and *ACS9* in response to eBL [48]. On the other hand, the role of BRs in establishing the cell fate in root epidermal cells has been suggested. For example, elevated levels of BRs and ethylene result in enhanced deposition of crystalline cellulose specifically in atrichoblasts, thus restricting cell expansion and overall root length [49]. BRs signaling has an important role in suppressing differentiation into trichoblasts and promoting non-hairy cell fate [50]. Finally, several studies of BRs receptor turnover specifically mention imaging to be performed on the trichoblast cell lines [50,51]. Observed preference in penetration only into the trichoblast cells may be the reason of lower biological activity of the compound comparing to the unlabeled eBL.

Next, we have confirmed that the effects of eBL-BODIPY on the physiology of arabidopsis roots mimic those of natural eBL. The pretreatment of seedlings by eBL impaired further eBL-BODIPY penetration inside the cells that might be a result of the limited receptor availability. Application of eBL-BODIPY impaired root elongation in a BAK1-dependent manner, confirming the specificity of the effect (Figure 1). This was observed both for the meristem length (impact on cell division) and cortical cell length (affected cell elongation). However, eBL-BODIPY did not cause “root waving”, an increase of the gravitropic curvature of the primary root, a phenotype connected with PIN2 relocalization and brassinosteroid-auxin signaling cross-talk [52], (Figure 1B). Waving phenotype is connected with BRs regulating polar auxin transport [53]. Indeed, the functioning of BRs in adjusting plant physiology is realized via a network of cross-talks with other phytohormones [54]. For example, BRs and auxins have been shown to act synergistically in the regulation of PIN2 turnover [55], in the maintenance of root apical meristem [56], and in the radial pattern formation of vascular bundles [57]. BRs promote the initiation of lateral root primordia in an auxin transport-dependent manner [58] by regulating the expression of *PIN* genes [59]. Moreover, auxins participate in the control of *BRI, BRL2* and *BRL3* expression, and may therefore influence brassinosteroid signaling capacity [60]. Genes responding both to BRs and auxin often contain regulatory element ARFAT [60]. In our study, both eBL and eBL-BODIPY induced transcription of *SAUR1A* and *GH3.3* (Figure 5), that is consistent with the data from full transcriptome profiling after short-term application of auxin IAA and BRs [59].

Especially strong is a connection of BRs with stress-related hormones, such as JA, ABA and SA (reviewed in [20]). The crosstalk has been implied at various levels, based on mutual regulation of hormone levels, sharing signaling components and downstream gene targets. For instance, BRs activate the production of secondary signaling messengers such as phosphatidic acid and Ca^2+^ and modulate the activity of antioxidant enzymes in tobacco [61]. In *A. thaliana* and rice, BRs increased the content of JA both under normal conditions and stress [62,63]; exogenous methyl jasmonate inhibited the expression of BRs biosynthesis genes and reduced the content of endogenous BRs in rice shoots [64]; BRs enhanced the JA-induced anthocyanin accumulation in leaves of Arabidopsis seedlings [65]. In our system, no significant changes were found in the transcription of *PDF1.2* and *LOX2* in response to any of the compounds. BRs co-operate with ABA in the regulation of developmental processes [66]. Indeed, BZR1 was found to directly bind to the promoter of *ABSCISIC ACID INSENSTIVE 5 (ABI5)*, an important transcription factor in ABA signaling [67]. Among the studied ABA marker genes, we found the induction of *CIPK20* by both eBL and eBL-BODIPY, while transcription of *LEA4-1, ABI1* was not affected. BRs treatment increased thermotolerance in wild-type *A. thaliana* seedlings, but not in *npr1-1* or *abi1* mutants that are impaired in SA or ABA signaling [68]. At the same time, 7 h of eBL application enhanced transcription of *PR1, PDF1.2* and *WRKY70* [68]. In our setup, eBL increased transcription of a batch of SA signaling genes (*PR1, PR2, PR5, WRKY70, WRKY38*), and a biosynthetic gene *ICS1*. eBL-BODIPY caused a similar effect on *PR1, PR5, WRKY70* and *ICS1*, although the induction levels were lower (Figure 5). 

To summarize, strong similarities in physiological and transcriptomic responses to eBL and eBL-BODIPY support our claim of analogous biological activity of the compounds. We thus propose eBL-BODIPY as a valid active probe to be used in the research of brassinosteroid perception and intracellular turnover.

## 4. Materials and Methods

### 4.1. Compounds

Synthesis and fluorescent properties of the 24-epibrassinolide conjugate (eBL-BODIPY) were previously described [40]. The 24-epibrassinolide (eBL) (Sigma Aldrich, St. Louis, MO, USA) and eBL-BODIPY were dissolved in DMSO; 1 mM stock solutions were stored at –20 ℃ in the dark. 

### 4.2. Plant Material

Experiments were performed on *A. thaliana* Col-0 as wild type (WT) and mutant lines *bak1-4* (SALK_116202). Seeds were surface sterilized by 1.6% sodium hypochlorite (30% of SAVO^®^, Unilever) solution with 0.02% (*v*/*v*) TWEEN20 (Sigma Aldrich, St. Louis, MO, USA). Seeds were stratified for 2 days at 4℃ in the dark. Seeds were germinated for 3 days in Petri dishes containing a half-strength Murashige–Skoog (½ MS) basal salt medium (Duchefa, Haarlem, Netherlands), pH = 5.7, supplemented with 1% (*w*/*v*) sucrose and 0.8% (*w*/*v*) plant agar (Duchefa, Haarlem, Netherlands) at 22 ℃ under long-day light (16 h light/8 h dark) conditions in a vertical position. Four days after germination, seedlings were transferred to Petri dishes containing the same medium supplemented or not with eBL/eBL-BODIPY and cultivated for 3 more days in a vertical position. Brassinosteroid solutions were prepared aseptically and added into warm media after autoclaving.

### 4.3. Root Growth Analysis

For the evaluation of primary root length, Petri dishes were scanned (Epson Perfection V700 Photo, Suwa, Japan). For the measurement of meristem and cortical cell length, roots were observed under an ApoTome Zeiss microscope with 10x objective in bright field settings. Images were analyzed by FiJi software [69]. At least 12 seedlings were measured for each variant. 

Localization of eBL-BODIPY was assessed by confocal microscopy (Zeiss LSM 880) with a 40×/63× C-Apochromat objective (NA = 1.2 W), excitation/emission wavelength was 495/505 nm. To visualize membrane fluorescence we used plants expressing PtdIns(4)P reporter, 2×mCherry_FAPP1-PH_. Cell walls were counterstained with 10 μg/mL propidium iodide [70].

### 4.4. Transcript Accumulation Measurement

For gene transcript quantification, five-day-old seedlings were aseptically transferred to Petri dishes containing a half-strength Murashige–Skoog medium containing 0.1/1 μM of eBL or eBL-BODIPY and incubated for 24 h at the same conditions as used for germination. Seedlings were pooled (cca 150 mg of fresh weight), flash-frozen in liquid nitrogen and stored at −80 °C. RNA was extracted as in [71]. Briefly, the tissue was homogenized in plastic Eppendorf tubes with 1 g of 1.3 mm silica beads using a FastPrep-24 instrument (MP Biomedicals, Solon, OH, USA). Total RNA was isolated using Spectrum Plant Total RNA kit (Sigma-Aldrich, USA) and treated with a DNA-free kit (Ambion, Austi, TX, USA). Subsequently, 1 μg of RNA was converted into cDNA with M-MLV RNase H–Point Mutant reverse transcriptase (Promega Corp., Madison, WI, USA) and an anchored oligo dT21 primer (Metabion, Planegg, Germany). Transcript levels were quantified by qPCR using a LightCycler 480 SYBR Green I Master kit and LightCycler 480 (Roche, Basel, Switzerland). The PCR conditions were 95 °C for 10min followed by 45 cycles of 95 °C for 10 s, 55 °C for 20 s, and 72 °C for 20 s. Melting curve analysis was then conducted. Transcript level values were normalized to TIP41 and analyzed using the “delta–delta Ct” method. A list of the analyzed genes and primers is available in Supplementary Table S1.

### 4.5. Data Processing and Analysis

All experiments were performed in at least three independent biological repetitions. Statistical analysis and graph preparation was performed using GraphPad Prism 8 software.

## 5. Conclusions

We present a detailed assessment of biological activity of eBL-BODIPY, a conjugate of brassinolide with the green fluorescent dye BODIPY. We show that eBL-BODIPY rapidly penetrates plant cell walls, and is perceived similarly as eBL. Based on competition assay with natural eBL, transcriptomic response and root phenotype, we conclude that the probe mimics effects of eBL and is suitable for short- and long-terms applications. We believe this compound has a high potential to be used in cell biology studies, especially to further dissect brassinosteroid-signaling pathways.

## Data Availability

Processed data is contained within the article and supplementary material. Raw data is available from the corresponding author upon request.

## Supplementary Materials

The following are available online at https://www.mdpi.com/article/10.3390/ijms22073599/s1. Figure S1: impact of eBL and eBL-BODIPY on meristem and cortical cell length. Figure S2: representative images of meristem of 7-day-old *Arabidopsis thaliana* Col-0 plants after 3 days of cultivation on media containing various concentrations of eBL-BODIPY. Figure S3: representative images of differentiation zone of 7-day-old *Arabidopsis thaliana* Col-0 plants after 3 days of cultivation on media containing various concentrations of eBL-BODIPY. Figure S4; impact of eBL and eBL-BODIPY on transcription of brassinosteroid biosynthetic gene *DWF4*. Table S1: primers used in this study. Video S1: intracellular localization of vesicles with eBL-BODIPY-associated fluorescence in differentiated trichoblast cells, Z-stack of 46 slices, scale bar 10 µm.
