# Supplementary material for: BODIPY Conjugate of Epibrassinolide as a Novel Biologically Active Probe for In Vivo Imaging
Source: Int J Mol Sci. 2021 Mar 30;22(7):3599. doi: 10.3390/ijms22073599 (PMC8036458; doi:10.3390/ijms22073599)
Supplement: Supplementary file 1 [file ijms-22-03599-s001.zip › Starodubtseva et al., 2021_Supplementary Materials_R1.pdf]

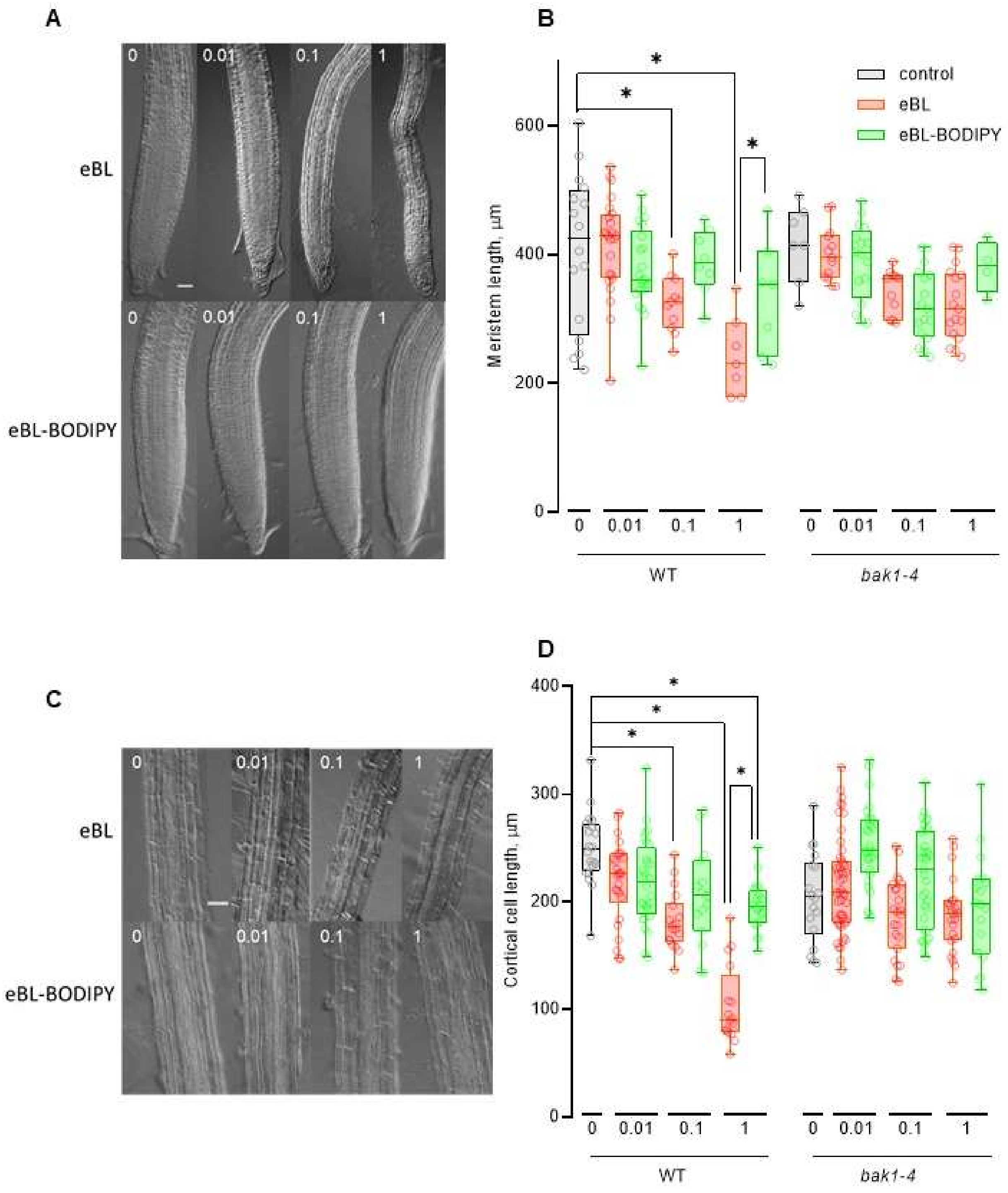

Figure S1. : Impact of eBL and eBL-BODIPY on meristem and cortical cell length (**a**) Representative images of WT meristem; (**b**) meristem length, n≥18; (**c**) representative images of WT cortical cells; (**d**) cortical cell length, n≥36; * - indicate significant differences between groups, one-way ANOVA.

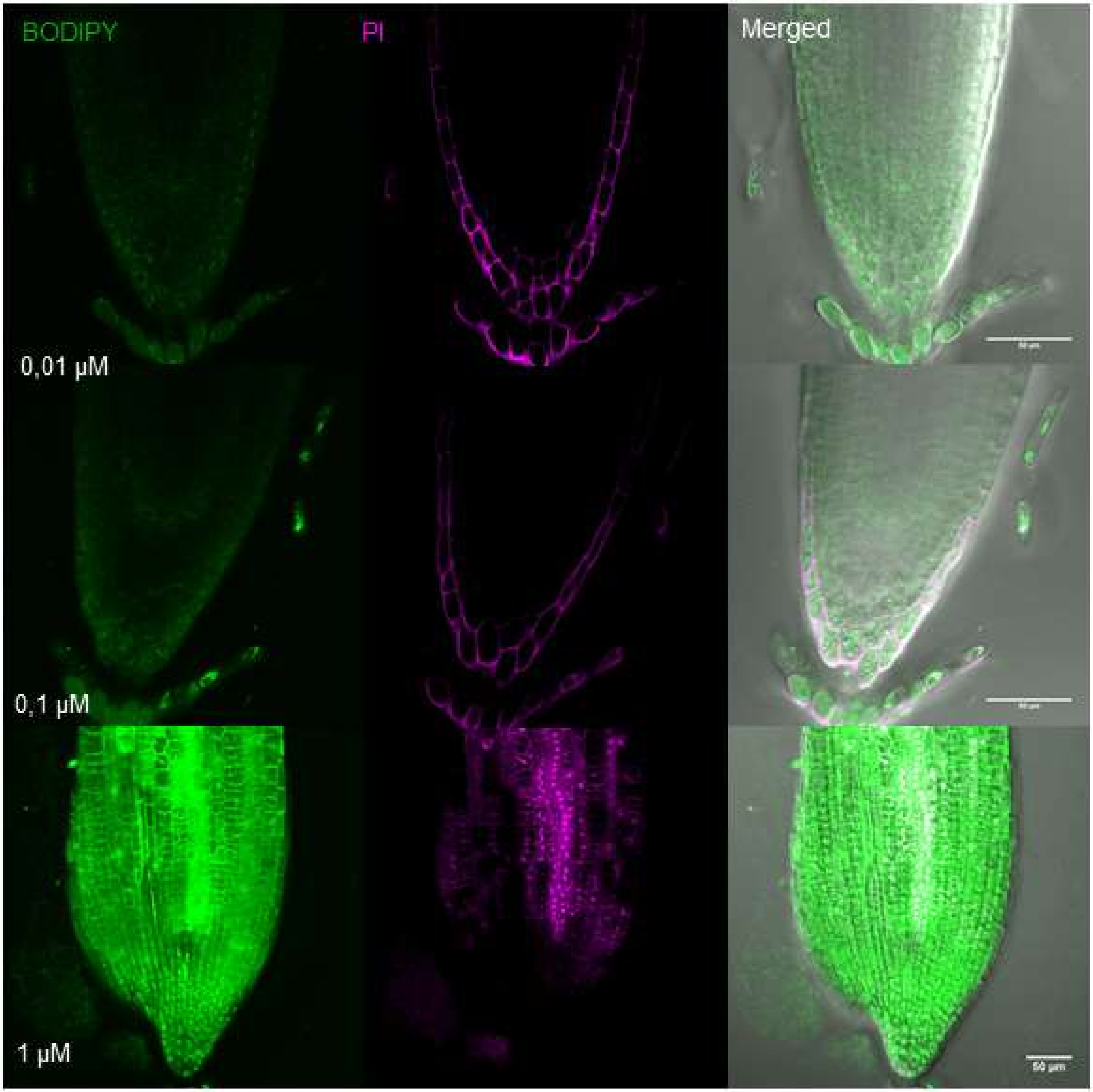

Figure S2. Representative images of meristem of 7-days old *Arabidopsis thaliana* Col-0 plants after 3 days of cultivation on media containing various concentrations of eBL-BODIPY; green - BODIPY fluorescence, magenta - propidium iodide (PI), merged - BODIPY + PI + bright field; scale bar 50 µm.

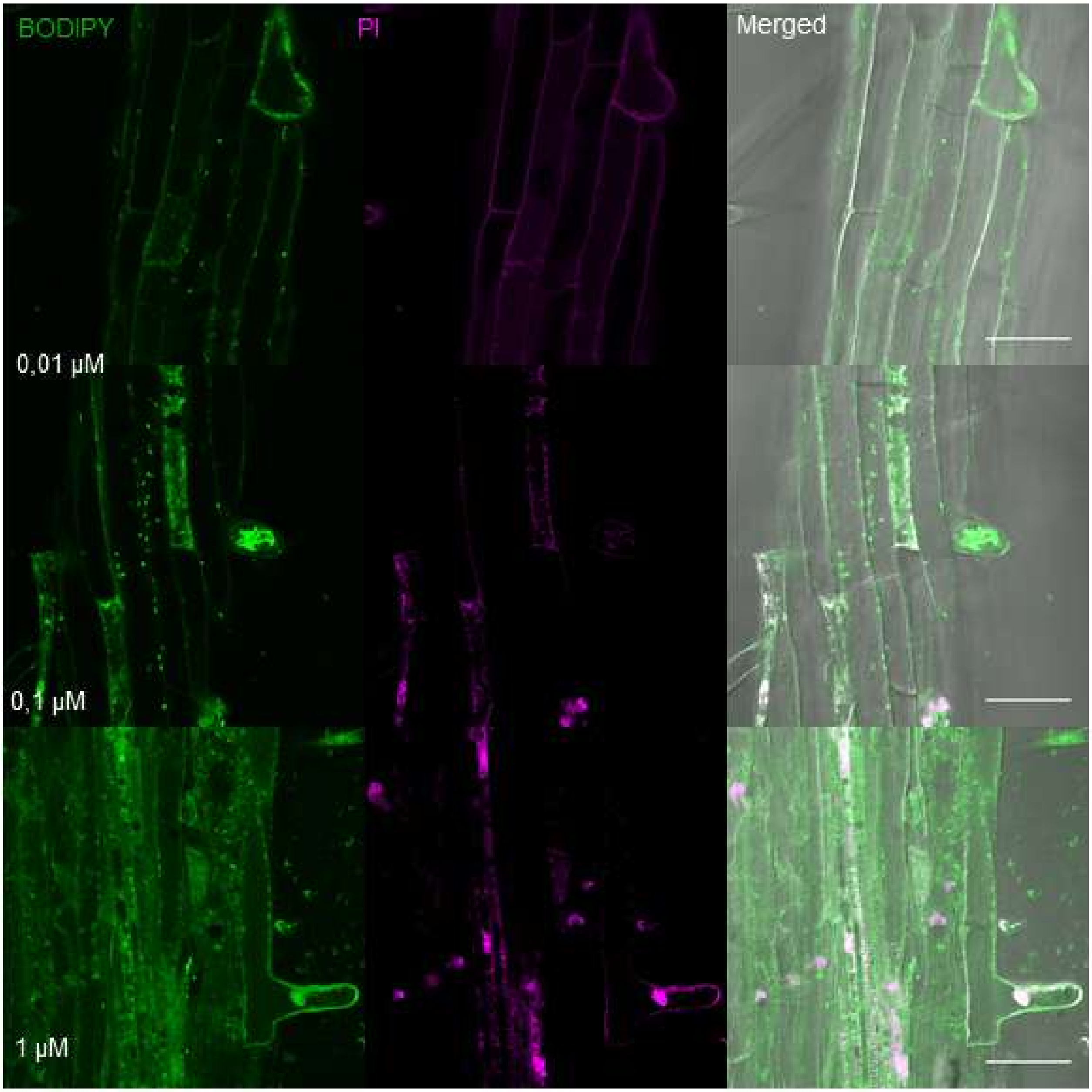

Figure S3. Representative images of differentiation zone of 7-days old *Arabidopsis thaliana* Col-0 plants after 3 days of cultivation on media containing various concentrations of eBL-BODIPY; green - BODIPY fluorescence, magenta - propidium iodide (PI), merged - BODIPY + PI + bright field; scale bar 50 um.

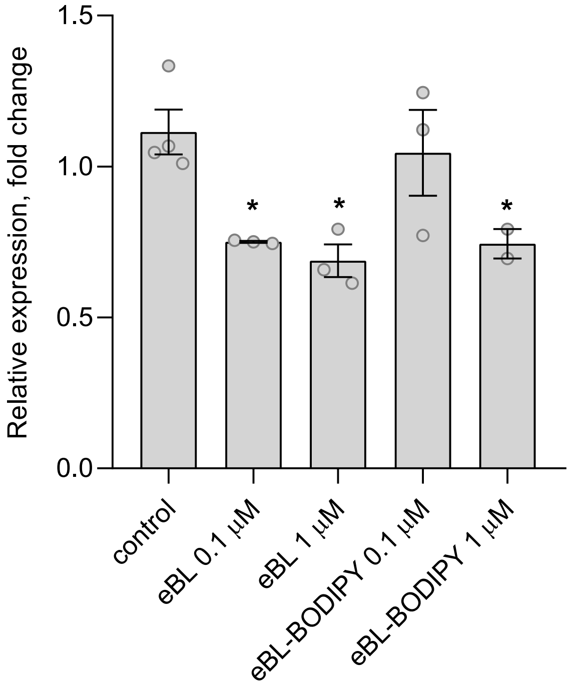

Figure S4. Impact of eBL and eBL-BODIPY on transcription of brassinosteroid biosynthetic gene *DWF4.* Five-days-old seedlings were exposed to 0.1 µM or 1 µM of eBL or eBL-BODIPY for 24 h prior to RNA extraction. Asterisks indicates variants significantly different from control, t-test, n=3-4, p<0.05.

Table ST1. Primers used in this study

Gene	Accession No	FP	RP
TIP41	AT4G34270	GTGAAAACTGTTGGAGAGAAGCAA	TCAACTGGATACCCTTTCGCA
AUX1	AT2G38120	ATTGACATTACCGTACTCGT	GTACTTCAAACCACCACTGAATGAC
SHY2	At1g04240	GGGCAAGATCTATGTTCATTGG	ACCTTTTGCCCTGTTTCTGA
SAUR1A	At4g38850	TTTGTTCTTCACAGTCACATCTCAG	ACAAGACACAAATGTATTAACATG
PR1	AT2G14610	AGTTGTTTGGAGAAAGTCAG	GTTCACATAATTCCCACGA
PR2	AT3G57260	TATAGCCACTGACACCAC	GCCAAGAAACCTATCACTG
ICS1	At1g74710	GCAAGAATCATGTTCCTACC	AATTATCCTGCTGTTACGAG
WRKY70	AT3G56400	CTTAATGCCAAATTCCCAAG	TGTGGTTTCCTATGTATGTG
WRKY38	At5g22570	GCCCCTCCAAGAAAAGAAAG	CCTCCAAAGATACCCGTCGT
LOX2	AT3G45140	ATCCCACCTCACTCATTACT	ATCCAACACGAACAATCTCT
PDF1.2	AT5G44420	ACGCACCGGCAATGGTGGAA	TGCATGATCCATGTTTGGCT
LEA4-1	AT1G32560	AGCAAAAGCTGATGAGAAGGCA	TAGGTCTGAGGAGGCACTGA
ABI1	AT4G26080	ATGTCGAGATCCATTGGCGAT	TTCCTTTCTCCGCTCATCCG
PR5	AT1G75040	TCTCCAGTATTCACATTC	CAATTCAAATCCTCCATC
GSTF12	AT5G17220	CATCTTCTTCGTCAGCCA	GTTGAAGTAATAGGTCTCCAC
GH3.3	AT2G23170	AACGGTTTGGTCTCAAGG	TGTCCCAGAGCTTGTGAG
CIPK20	AT5G45820	AAATCATGGAGAATT	CTTTCTCAAACAAAC
DWF4	AT3G50660	AACAGACGATGATCTTTTGGG	CTTCAACGGCTTTAGGGCAA
